# Development, identification and validation of CAPS marker for SHELL trait which governs dura, pisifera and tenera fruit forms in oil palm (*Elaeis guineensis* Jacq.)

**DOI:** 10.1371/journal.pone.0171933

**Published:** 2017-02-13

**Authors:** B. Kalyana Babu, R. K. Mathur, P. Naveen Kumar, D. Ramajayam, G. Ravichandran, M. V. B. Venu, S. Sparjan Babu

**Affiliations:** 1 ICAR-Indian Institute of Oil Palm Research, Pedavegi, West Godavari (Dt), Andhrapradesh, India; 2 KL University, Guntur (Dt), Andhra Pradesh, India; National Institute of Plant Genome Research, INDIA

## Abstract

The oil palm fruit forms (dura, pisifera and tenera) governed by the shell thickness gene (*Sh*) plays a major role in identification of fruit type and also influences palm oil yield. Identification of desired fruit type is a major asset to the breeders and oil palm workers for applications in breeding, seed certification and to reduce time, space and money spent on identification of fruit form. In the present study, we developed *Sh* gene specific primer pairs and bulk segregant analysis was done using 300 genomic and 8 genic SSR markers. We identified one cleaved amplified polymorphic site (CAPS) marker for differentiation of oil palm fruit type which produced two alleles (280 and 250bp) in dura genotypes, three alleles in tenera genotypes (550, 280, and 250bp) and one allele in pisifera genotypes (550bp). The shell allele sequencing results showed that two SNPs were present, of which SNP2 contributed for variation of fruit forms. The nucleotide ‘A’ was present in only dura genotypes, where as ‘T’ was present only in pisifera genotypes, which in turn led to the change of amino acid lysine to aspargine. The identified CAPS marker was validated on 300 dura, 25 pisifera and 80 tenera genotypes, 80 dura/ pisifera cross progenies and 60 lines of tenera/ tenera cross progeny. Association mapping of marker data with phenotypic data of eight oil yield related traits resulted in identification of seven significant QTLs by GLM approach, four by MLM approach at a significant threshold (P) level of 0.001. Significant QTLs were identified for fruit to bunch and oil to bunch traits, which explained R^2^ of 12.9% and 11.5% respectively. The CAPS marker used in the present study facilitate selection and timely distribution of desirable high yielding tenera sprouts to the farmers instead of waiting for 4–5 years. This saves a lot of land, time and money which will be a major breakthrough to the oil palm community.

## Introduction

Oil palm (*Elaeis guineensis* Jacq.) belongs to the family *Arecaceae* which contributes nearly 40 percent of edible vegetable oil production throughout the world [1]. The palm oil production is five times more the than the annual oil yielding crops [2]. Indonesia is the largest producer of palm oil followed by Malaysia; however India is at its lag phase of growth in palm oil production. The oil palm genotypes are divided into dura, pisifera and tenera forms based on the shell thickness, which is a monogenic and co-dominantly inherited trait. With the discovery of SHELL thickness gene (*Sh* gene) by the oil palm researchers in the Congo during 1940s led to more focus on increasing the oil palm production [3]. The dura (D) genotypes consist of thick shell (Sh/Sh, dominant homozygote), whereas pisifera (P) genotype has shell less with recessive homozygous sh/sh allele [4]. The tenera (T) genotype has thin shell which has 30% more mesocarp and oil production than dura and pisifera, which is generally produced as hybrid from cross between D and P. The tenera hybrid yields more oil and also is the basis for commercial palm oil production in all the oil palm growing parts of the world. Identification of these three fruit forms is a challenging task for oil palm breeders and growers. However, the fruit form determination can be possible only after 4–5 years by dissection of the fruit based on the thickness of shell and fibre ring, which requires a lot of time and space.

With the advancement of molecular marker technology, a lot of progress has been made in the oil palm molecular biology. The first step towards use of molecular markers in oil palm was started by Mayes et al. [5] where they carried out RFLP based genetic mapping. Molecular markers such as RAPD [6], AFLP [7], and SSR [8] were used for genetic diversity [9], linkage mapping [10], and association mapping [11] and for linkage map construction studies [8]. The 1.535 Gb of the *E*. *guineensis* (AVROS, pisifera fruit form) reference genome assembly were released to public in 2013 [12]. Ting et al. [13] developed high density SNP array and SSR based genetic maps in oil palm hybrids. Singh et al [14] found two independent mutations in the MADS box transcription factor of SHELL gene which is homologous to gene SEEDSTICK (STK) gene in *Arabidopsis thaliana* which controls the ovule identity by homozygosity mapping. They also found one SNP in the 28 or 30 codon which impairs the normal DNA binding of shell (*Sh*) which leads to shell less phenotype. Satish and Mohan Kumar [6] identified RAPD markers for identification of parental and hybrid genotypes.

Identification of robust and simple molecular marker to distinguish the three fruit forms is a challenging task for oil palm breeders and growers. Because, it will be useful to diagnose the fruit form at seedling stage instead of waiting for 4–5 years by traditional means. It is also useful in certification of high yielding seed production by selective breeding of DxP crosses, particularly on plantation that lack stringent quality control or where natural pollination with small quantities of tenera or dura pollen can occur. However from India, there were no reports on the identification and validation of robust *Sh* allele specific marker among the Indian oil palm genetic background. With this, in the present study we aimed at identification of suitable molecular markers for differentiation of dura, pisifera and tenera genotypes at the seedling stage. The objectives of the study include 1) identification of suitable molecular marker for differentiation of dura, pisifera and tenera genotypes, 2) Association mapping analysis of important oil yield related traits and 3) validation and marker assisted selection for shell thickness among the African and Indian oil palm germplasm.

## Materials and methods

### Plant materials and genomic DNA isolation

In the present study, 300 dura and 25 pisifera genotypes and 80 tenera progeny lines obtained from DxP cross, and 100 TxT cross progeny were used for genotyping, validation and confirmation of the SSR markers. The oil palm plantations and nursery were raised at ICAR-Indian Institute of Oil palm Research (IIOPR), Pedavegi, India (latitude 16° 48’N, longitude 81°7’E). The experiments were conducted with the consent of Director, ICAR-IIOPR, India. For bulk segregant analysis (BSA), 10 each of dura and pisifera genotypes was used (Table 1). The genotypes for confirmation of the marker were selected based on their fruit phenotyping. The palms selected under the study were at 10–15 years of age, while the progeny were at 4–5 year old stage. The genomic DNA of oil palm genotypes was isolated by standard method as described by Murray and Thomson [15] with few modifications such as repetition of chloroform: iso-amyl alcohol step to achieve good quality of DNA. The quality and quantity of genomic DNA was checked on 0.8% agarose gels along with uncut lamda DNA as a control. The DNA samples were normalized to a uniform concentration (25ng/μl) for SSR genotyping. For BSA the genomic DNA of ten each dura and pisifera genotypes were bulked respectively.

**Table 1:** The genotypes selected for bulk segregant analysis.

S. No	Dura	Pisifera
1	CD409	P195
2	CD100	P78
3	CD540	P110
4	CD45	P14
5	CD166	P76
6	CD83	P77
7	CD45	P75
8	CD194	P13
9	CD108	P17
10	CD206	P30

### Fruit phenotyping

Fruit phenotyping was carried out in 12–15 year old plantations. A random set of fruits from five bunches were harvested from known dura, pisifera and tenera genotypes (for validation of identified marker) and fruit form was determined as per the earlier reports [16].

### *In-Silico* mining of SSRs from SHELL and homologous genes

The EST sequences of oil palm MADS-box transcription factor, and *Arabidopsis thaliana* MADS box transcription factor like AGL11 were down loaded in FASTA format from NCBI website (Table 2). The downloaded sequences were obtained in FASTA format for sequence assembly and SSR analysis. The online software tool “Websat” [17] was used for identification of microsatellite repeats and primer designing which uses the primer 3 software. Six classes of SSRs, *i*.*e*., mono-, di-, tri-, tetra-, penta-, and hexa nucleotide repeats were targeted for identification of SSRs using this tool. The search criteria used for identification of EST-SSRs was as follows. The minimum number of repeats as 10 for mononucleotide, 6 for di nucleotide, 4 for tri nucleotide, and three for each tetra, penta and hexa nucleotides. The main parameters for primer designing were, GC content of 40–60%, annealing temperature (Tm) of 50–60°C and expected amplified products size of 100–450 bp. All other parameters were set to default values.

**Table 2:** The EST based SSR markers identified and developed.

Primer Name	Sequence	Repeat motif	Expected product size	Gene from which marker developed	GenBank Accession number	Length of nucleotides	Annealing temperature
EG01F	5′TGCCTTGTCTCTCCAGAAAACT3′	(AAAG)3	384	*Arabidopsis thaliana* chromosome 4		22	60.415
EG01R	5′CACTGATGCACATGAAACTGAA3′					22	59.764
EG02F	5′TCTCTTCTCGAAACTAAGCATGG3′	(CTT)4	397	do		23	60.028
EG02R	5′CATCCCGTGTACTATCTCCCTC3′					22	59.848
EG03F	5′CCCGAGAAAGTTGGATACAGAA3′	(T)10	279	do		22	60.474
EG03R	5′AACGTCACTTGTCGATTTGTTG3′					22	60.074
EG04F	5′TGAAATCGAAAACGCGCAGA3′			*Arabidopsis thaliana* agamous-like MADS-box protein AGL11 mRNA	NM_117064.6	20	59
EG04R	5′CCAGATCCAGAACCAGCAGT3′					20	59
EG05F	5′GTGTCCGATCCAAGAAGCAC3′			*Arabidopsis thaliana* agamous-like MADS-box protein AGL5 mRNA	NM_180046.2	20	60
EG05R	5′CCCGACTGGTGAGAAGAAGT3′					20	60
EG06F	5′CAATGGAGGAAGGTGGGAGT3′			*Arabidopsis thaliana* agamous-like MADS-box protein AGL1 mRNA	NM_115740.2	20	59
EG06R	5′CGGCATCACACAAGACAGAG3′					20	59
EG07F	5′GGAATTTGCGGTGTTGGACT3′			*Elaeis guineensis* MADS-box transcription factor 21-like (LOC105034563), mRNA	XM_010909778.1	20	59
EG07R	5′GGCAACCTCAGCATCACAAA3′					20	59
EG08F	5′GGAATTTGCGGTGTTGGACT3′			do	do	20	59
EG08R	5′TATCTGGTGGCGCAACTTTG3′					20	59

### SSR marker analysis

The genomic SSRs used for genotyping and bulk segregant analysis were obtained from the earlier reports [8]. The bulk segregant analysis was performed with 300 genomic and 8 genic SSR markers on bulk DNA of dura and pisifera genotypes. The thermal reactions were performed in 25 μL reaction volume containing about 25–40 ng of template DNA, 2 μL of 10X buffer having 15 mM MgCl2, 0.2 μM of each forward and reverse primer, 2 μL of 2 mM dNTPs, 0.2 μL of 1 U of *Taq* DNA polymerase (Invitrogen, USA). The amplifications were performed in a Thermo cycler (MJ Research, USA) programmed for an initial denaturation of 3 min at 95°C followed by 35 cycles of 30 s at 95°C, 30 s of 45°C annealing temperature, extension of 1.0 min at 72°C, with a final extension of 10 min at 72°C, and final hold at 4°C.

### Nucleotide sequencing of SHELL alleles and bio-informatics analysis

The alleles obtained from the thermal cycler were sequenced by out sourcing to the BioServe (India) private limited. In brief, the PCR products were purified using a QIAquick PCR purification kit (Qiagen Inc., Valencia, CA, USA) according to the manufacturer’s protocol. Sequence editing and assembly of the contigs were performed using Sequencher 4.10. For comparisons among the sequences of genotypes, the ClustalW and Clustal Omega online tools with multiple alignment option were used and adjusted manually by the authors. The PCR products were sequenced from both ends and the resulting termination products were analyzed on an ABI 3130XL Genetic Analyzer. The two resulting sequence traces derived from opposite ends of each amplicon were analyzed, aligned with standard DNA analysis software Phred and Phrap (http://www.phrap.org/). The nucleotide sequences were translated into a protein sequences using the online tool Expasy (web.expasy.org/translate/) and alignment was done using Clustal omega online tool. The nucleotide and protein sequences obtained from dura and pisifera genotypes were analyzed using blastn search for identification of homologous sequences in oil palm and other crops at an E-value of more than 2e-13.

### Restriction site analysis

The *Sh* allele sequences of the dura, pisifera and tenera were screened for potential restriction sites in the polymorphic regions using the online available tool restriction mapper (http://www.restrictionmapper.org). Ten μL of the PCR product obtained in the amplification with SHELL gene specific primer were digested with 10 U of different restriction enzymes (Genetix, USA) along with given specific buffer. Digestion was performed overnight at 37°C and also digestion at 37°C performed for 2 hours which gave similar results. Restriction fragments were visualized by electrophoresis as described above.

### Association mapping and data analysis

Briefly, a total of 100 oil palm germplasm representing five African countries (Cameron, Tanzania, Zambia, Nigeria and Guinea Bissau) were used for association mapping studies. The palms are at 15 years age, maintained at experimental field of ICAR-Indian Institute of Oil Palm Research, Andhra Pradesh, India. The seedlings were raised in a triangular system of 9 x 9 x 9m spacing. The phenotypic data was characterized for eight oil yield related parameters (bunch weight, BW; fruit to bunch ratio, FB; mesocarp to fruit, MF; kernel to fruit, KF; shell to fruit, SF; oil to dry mesocarp, ODM; oil to wet mesocarp, OWM; and oil to bunch, OB). The phenotypic data used was collected from the average of four years (2009–13). Fifty SSR markers were used for genotyping and association studies. The primer sequences were obtained from the Malaysian Palm Oil Board (MPOB) website (http://www.mpob.gov.my). Association analysis was done by using phenotypic data of 100 oil palm germplasm, genotypic data of 50 SSR markers and population structure data (Q matrix) by using software TASSEL [18]. The marker–trait association analysis was conducted using TASSEL 3.0 software along with the general linear model (GLM) and mixed linear model (MLM) procedures. The kinship matrix was used in addition to the genotypic, phenotypic and Q matrix data in the MLM approach. The significant threshold for the association was set at P, 0.01 and 0.001.

## Results and discussion

In order to find the markers linked to the SHELL thickness in oil palm, in the present study a three tier approach has been used. We initially downloaded EST sequences of oil palm MADS-box transcription factor like genes, and *Arabidopsis thaliana* MADS box transcription factor genes like AGL11 in FASTA format from NCBI website and designed eight gene specific SSR markers. Then bulk segregant analysis was performed among the bulk DNA of dura and pisifera genotypes and association mapping was done to identify the significant QTLs. We further analyzed next generation sequencing analysis of the SHELL gene amplified products.

### *In-Silico* mining of EST based SSRs

The *SHELL* gene is responsible for identification of oil palm fruit forms *viz*., dura, pisifera and tenera [3]. Identification of desired fruit form at the seedling stage saves the time period of 4 to5 years, space and ensures supply of desired tenera sprouts to farmers which eliminate contamination of dura palms. Hence, there is a need to identify a suitable molecular marker for identification of these three fruit forms at seedling stage. It was also reported that SHELL gene function and expression was highly conserved among the higher plant species [14]. In *Arabidopsis*, shatterproof (*SHP1*) and seed stick (*STK*) genes are controlled by MADS-box transcription factor which controls differentiation of ovule and seed [19, 20]. Similarly in tomato, SHP homologues were present which controls fleshy fruit expansion [21]. With this aim, as a preliminary study we developed eight EST based microsatellite primers from oil palm MADS box transcription factor like, and *Arbidopsis thaliana* agamous like MADS box AGL11, AGL5, AGL1 mRNA sequences (Table 2). The primer pairs (Eg 01- Eg 03) were homologous to the SHELL specific region of *A*. *thaliana* chromosome 4 genomic region. The primer pairs (Eg 04 –Eg 06) were developed from *A*. *thaliana* MADS box transcription factor AG11, AG5 and AG1 like mRNA sequences respectively. The primer pairs Eg 07 and Eg 08 were developed from the *Elaeis guineensis* MADS box transcription factor like genes. Primer Eg SHP was obtained from the earlier studies [14]. Very limited publications were available on the development of shell specific primers [22, 23]. Ting et al [24] did mining of EST based SSRs of oil plam expressed sequence tag sequences and found to contain 722 SSRs with a variety of motifs and di nucleotide repeats formed the largest group.

### Bulk segregant analysis

**Fig 1:**
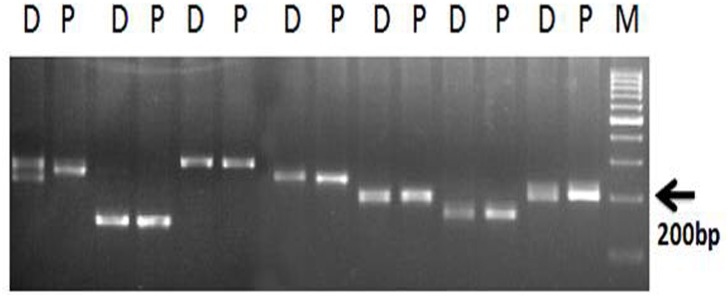
The bulk segregant analysis of dura and pisifera genotypes using microsatellite markers (D – dura, P- pisifera, M – 100bp marker.

Bulk segregant analysis (BSA) was performed with the 300 genomic SSR markers and eight EST-SSRs. The genomic DNA of ten each dura and pisifera genotypes were bulked for BSA analysis. Out of the 308 SSR markers, 30 markers were found to be polymorphic between dura and pisifera bulk genomic DNA. However, we did not find any clear cut differentiation among the individual dura and pisifera genotypes. The polymorphism pattern of bulk DNA of D and P genotypes are given in Fig 1. Out of the 8 EST based SSRs, three were polymorphic, whereas remaining were mono-morphic. The results from BSA showed that no marker was found to differentiate the individual dura and pisifera genotypes.

### Identification of CAPS marker

**Fig 2:**
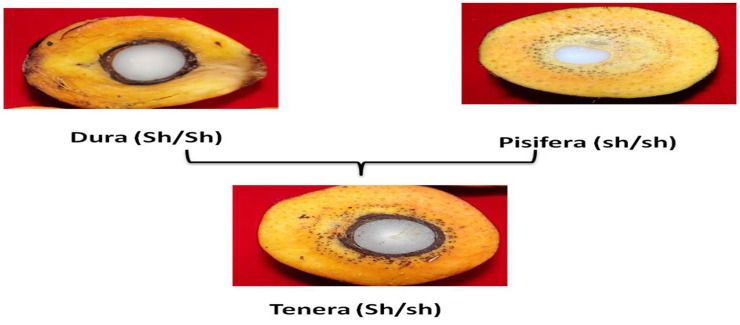
The production of tenera genotypes obtained from the cross between dura and pisifera genotypes.

The oil palm fruit form governed by shell thickness gene (*Shell* gene) distinguishes the oil palm genotypes as dura, pisifera and tenera fruit forms. The dura genotype has thick shell, consisting of dominant Sh allele (Sh/Sh), whereas pisifera genotypes are shell less, consisting of recessive shell alleles (sh/sh) [4]. However, the tenera genotypes considered as hybrids, which have heterozygous Sh alleles (Sh/sh) derived from the cross between dura and pisifera (Fig 2). Hence, the tenera genotypes were considered to be more profitable to the farmers. The identification of fruit form has several advantages like in certification of high yielding seed production by selective breeding of D X P crosses. It also useful to identify the true female sterile pisifera palms, and also ensures supply of desired tenera sprouts to the oil palm growers instead of waiting for 4–5 years.

**Fig 3:**
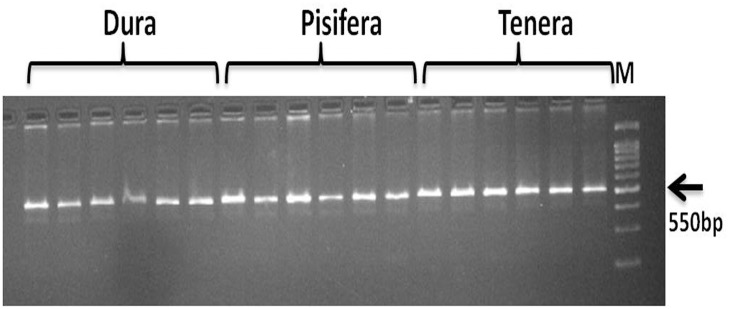
The agarose gel based banding pattern of dura, pisifera and tenera genotypes using EgSHP marker.

Earlier research findings [14] found two independent mutations in the DNA binding domain of homologue of MADS box transcription factor. The SHELL gene was localized via homozygosity mapping to a specific region on the 4.3 Mb scaffold 43 (p^3^ sc00043), which was mapped to chromosome 2. Then we selected SHP specific primer sequences (EgSHP-Forward-TTGCTTTTAATTTTGCTTGAATACC, reverse -TTTGGATCAGGGATAAAAGGGAAG) to amplify the shell gene which was unique in the reference pisifera genome [14]. The primer itself did not show any polymorphism between dura, pisifera and tenera genotypes and it can’t be used to identify oil palm fruit types (dura, pisifera, and tenera). The SSR marker EgSHP generated product with an amplification size of 550bp allele in the dura, pisifera and tenera genotypes (Fig 3).

### *Sh* allele sequence analysis

**Fig 4:**
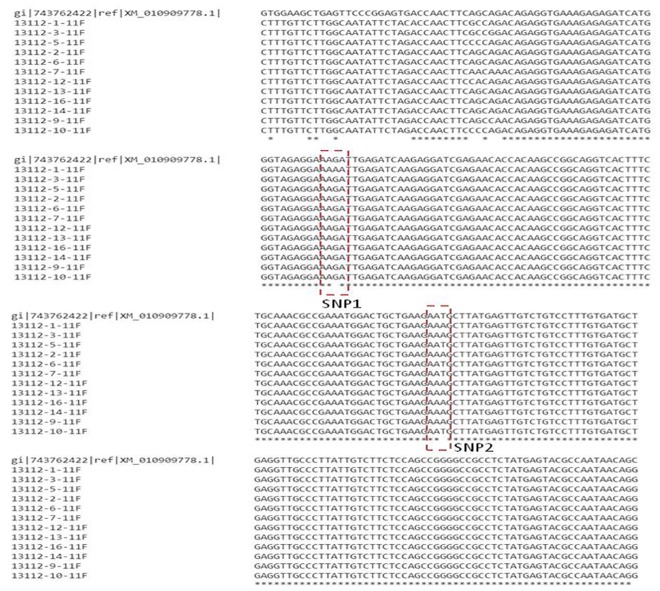
The multiple sequence alignment of nucleotide sequences of the Sh alleles of selected dura, pisifera and tenera genotypes along with the oil palm MADS box transcription factor-21 like mRNA sequence (13112–1, 2 and 3 – dura, 13112–5, 6 and 7 – pisifera and 13112–9, 10, 12–14, and 16 – tenera).

The 550bp alleles amplified from the selected dura, pisifera and tenera genotypes were sequenced for further analysis and deposited in NCBI (KX465097-KX465106, KX447588, and KX363799). The nucleotide sequences were blastn searched for finding the homologous sequences. The nucleotide sequences showed homology to MADS box transcription factor-21 like mRNA sequence (GenBank accession no. XM_010909778.1) at an E-value of 4e-13. The nucleotide sequences were aligned using the multiple sequence alignment option of Clustal omega online tool and ClustalX stand alone software. The alignment results showed that there were two single nucleotide polymorphisms (SNPs) existed across the sequences along with oil palm MADS box transcription factor-21 like mRNA sequence (Fig 4). The first SNP was present at 169^th^ nucleotide position which was found to be transition (G to A). The nucleotide ‘G’ was present in all genotypes, whereas ‘A’ was present in only single dura genotype CD100. Hence, this SNP may not be contributing variation for differentiation of dura, pisifera and tenera genotypes. The second SNP (transition from A to T) was found at nucleotide position of 248. The nucleotide ‘A’ was present only in dura genotypes, whereas ‘T’ nucleotide was present only in pisifera genotypes. The tenera Sh allele sequences found to contain either A or T nucleotide as expected. From the above results it was clear that the second SNP was contributing for variation in the fruit forms of dura, pisifera and tenera genotypes. Similar results were also obtained in the earlier reports by Singh et al [14], where they found two independent mutations in the MADS box transcription factor of SHELL gene which is homologous to gene SEEDSTICK (STK) gene in *Arabidopsis thaliana* which controls the ovule identity by homozygosity mapping. They also found one SNP in the 28 or 30 codon which impairs the normal DNA binding of shell (*Sh*) which leads to shell less phenotype. However, in the present study only one SNP contributing to fruit form and this is likely due to the limited genetic background used in this study.

**Fig 5:**
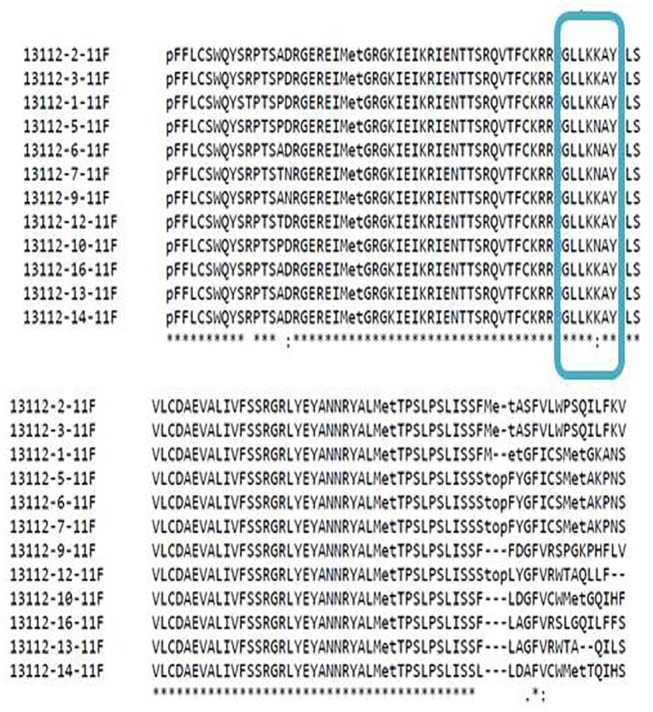
The multiple sequence alignment of protein sequences of the Sh alleles of selected dura, pisifera and tenera genotypes along with the oil palm MADS box transcription factor-21 like mRNA sequence (13112–1, 2 and 3 – dura, 13112–5, 6 and 7 – pisifera and 13112–9, 10, 12–14, and 16 – tenera).

**Fig 6:**
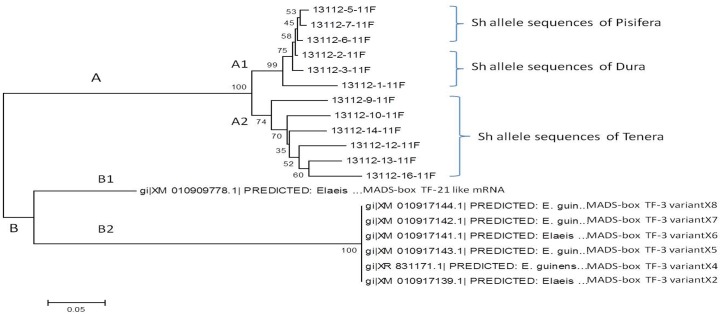
MEGA 4 clustering analysis of Sh allele sequences obtained in the study along with homologous sequences obtained from BLASTn search.

The nucleotide sequences were further analyzed by translating into protein sequences using the expasy online tool. The protein sequences were also aligned using the multiple sequence alignment option of clustal omega (Fig 5). The amino acid change also observed at the first SNP position *i*.*e*., from SWQYS(**R)**PT to SWQYS(**T)**PT (from arginine to threonine). Only one dura genotype consisted of arginine and its role need to be studied. The dura genotypes had the amino acid composition of GLLKKAY at the second SNP position, whereas pisifera genotypes had the amino acid composition of GLLKNAY. There was a change of amino acid from Lysine (K) to Aspergine (N) amino acid (Fig 5). The tenera genotypes consisted of both amino acid patterns of either GLLKKAY or GLLKNAY. The nucleotide and amino acid composition of dura, pisifera and tenera Sh alleles was given in Table 3. The nucleotide sequences were further analyzed by MEGA4 software for construction of phylogeny of shell sequences obtained in the study along with homologous sequences. The nucleotide sequences were grouped into two major clusters A and B (Fig 6). The major cluster A consisted of *Sh* allele sequences obtained from our study, where as cluster B consisted of homologous sequences belongs to the *E*. *guineensis* MADS-box transcription factor mRNA sequences. The cluster A further divided into two sub-clusters A1 and A2. The sub-cluster A1 comprised of dura and pisifera *Sh* allelic sequences, where as A2 comprised of tenera *Sh* allelic sequences. Under the sub-cluster A1, further grouping was done according to the oil palm fruit form, i.e., dura and pisifera. The clustering pattern obtained from the MEGA4 software clearly differentiated the Sh allele sequences based on the fruit form of oil palm i.e., dura, pisifera and tenera. The results showed that Sh allele is responsible for the fruit form of oil palm genotypes (dura, pisifera and tenera). However, the homologous sequences formed a separate cluster (B) which further divided into two sub-clusters B1 and B2. The B1 cluster consisted of single sequence (GenBank accession No. XM_010909778.1) which denotes *Elaeis guineensis* MADS-box transcription factor 21-like (LOC105034563), mRNA. It was also found that this transcription factor sequence also had the two SNPs, of which one was responsible for fruit form differentiation in oil palm (Fig 4). This sequence also found to be more homologous with the *Sh* allele sequences of our study than other sequences. The cluster B2 consisted of *Elaeis guineensis* MADS-box transcription factor 3 like mRNA sequences (accession nos. XM_010909778.1, XR_831171.1, XM_010917144.1, XM_010917143.1, XM_010917142.1, XM_010917141.1 and XM_010917139.1) which were of different variants such as X4 to X8 and X2. The phylogenetic analysis gave a clear justification of differentiation of the *Sh* allele sequences according to their fruit form and also variation of transcription factor variants.

**Table 3:** The nucleotide and amino acid composition of dura, pisifera and tenera genotypes at the SNP2 position of *Sh* alleles.

Marker	Dura allele	Pisifera allele	Tenera allele
Nucleotides	GGA CTG CTG AAG AA**A** GCT TAT	GGA CTG CTG AAG AA**T** GCT TAT	Either GGA CTG CTG AAG AA**A** GCT TAT/ GGA CTG CTG AAG AA**T** GCT TAT
Amino acids	Gly Leu Leu Lys Lys Ala Tyr (GLLK**K**AY)	Gly Leu Leu Lys Asn Ala Tyr (GLLK**N**AY)	Either GLLKKAY or GLLKNAY
Hind III restriction site	-	5’ AA **A** GCT TAT 3’	-

### Identification of restriction sites to amplify the SNP2 containing region

**Fig 7:**
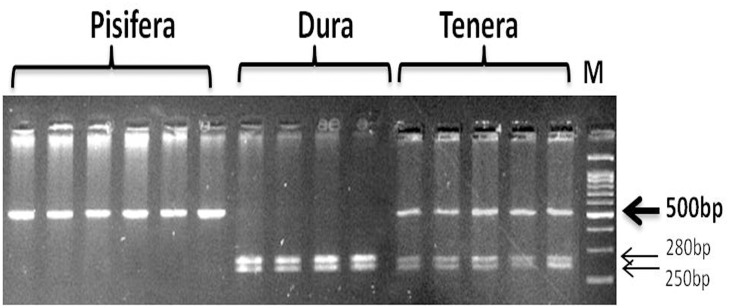
Identification and differentiation of dura, pisifera and tenera genotypes using the CAPS marker (D- dura, P- pisifera, T- tenera).

Since, we obtained information regarding availability of SNP variation between dura and pisifera genotypes, we analyzed sequences to find the right restriction sites available in the shell alleles of dura and pisifera genotypes. The online tool ‘restriction mapper’ was used to find the restriction sites among the nucleotide sequences. We tried five restriction enzymes *viz*., BseMII, HindIII, MboII, EcoP15I, and NaeI which were at the nucleotide positions of 259, 280, 266, 221 and 216 respectively near to the SNP2. The restriction digestions were performed for different temperatures and time suitable for different restriction enzymes. However, only Hind III restriction enzyme which has recognition site of AAGCTT near to the second SNP could able to produce significant differential alleles. The PCR amplified product (550bp) was digested with the Hind III restriction enzyme for 18 hours at 37°C and also for 2 hours at 37°C. We got similar results in both the cases. The Hind III digested PCR product produced two alleles (280bp and 250bp) in dura genotypes, three alleles in tenera genotypes (550bp, 280bp, and 250bp) and one allele in pisifera genotypes (550bp) (Fig 7). As expected, the tenera genotypes showed the hybrid allelic pattern since it was derived from cross between dura and pisifera genotypes. Singh et al [14] placed the *Shell* gene locus in T128 linkage group 7 and mapped by sequence similarity to 3.4Mb of scaffold 43 at the end of chromosome 2. In the present study, we identified the primer sequence at the upstream and downstream region of SHP1 region of Shell gene which uniquely amplified the dura, pisifera and tenera genotypes.

### Validation and marker assisted selection of CAPS marker across populations

**Fig 8:**
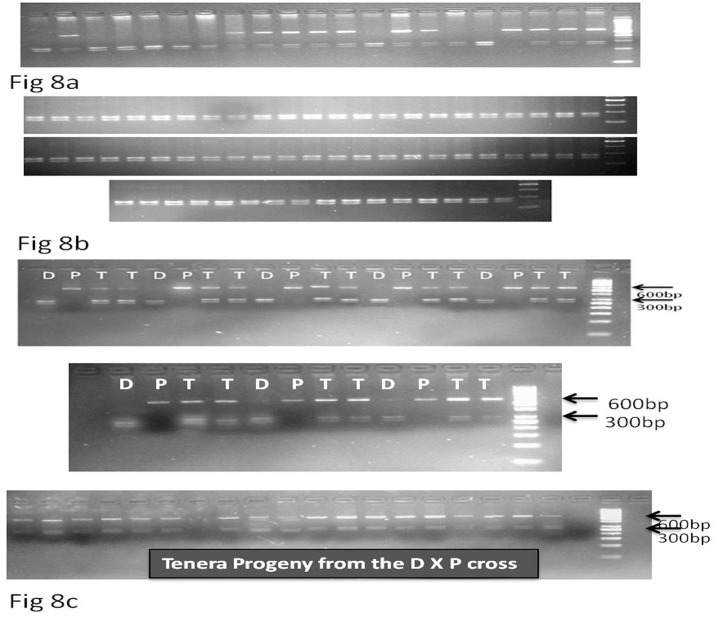
Validation and marker assisted selection of dura, pisifera and tenera genotypes using the identified CAPS marker (D- dura, P- pisifera, T- tenera) (Fig 8a. T X T cross progeny; Fig. 8b. Dura genotypes from African germplasm; Fig. 8c Tenera genotypes obtained from the D X P cross).

Validation and confirmation is required for any marker assisted programmes for identification of desired germplasm or genotypes. With this aim, the identified CAPS marker was tested on 300 dura genotypes available in our African germplasm collection experimental field, 25 pisifera genotypes, and 80 tenera genotypes. All the results showed clear cut differentiation of dura, pisifera and tenera genotypes using the CAPS marker as shown in Fig 7. To further validate the CAPS marker, we tested on 80 DXP cross progeny lines (Fig 8c), 60 lines of TxT cross progeny (Pisifera improvement block) (Fig 8a). All the results confirmed that the tenera genotypes had allele from both the dura and pisifera genotypes as expected and could differentiate the dura and pisifera fruit forms (Fig 8). The results of the study differ from the earlier reports, which showed the robustness of the present study. Ritter et al [22] used three primers for differentiation of dura, pisifera and tenera forms and the PCR protocol was different for tenera genotypes in comparison to remaining two types of fruit forms. However, in the present study we used a simple PCR protocol for the three fruit forms. In their study they used three primers, however in our study we used only single CAPS marker to differentiate the three fruit forms. The percentage of purity in tenera seeds was not determined in their study, and they suggested that it could be determined using DNA from embryos like several crops. However, in the present study we already conducted the purity testing of the tenera seeds by extraction of DNA from the embryos and determined their fruit form as given in the above protocol. Recently Reyes et al [23] developed *Sh* allele specific PCR tool for the differentiation of these three fruit forms. However, they validated and did blind trial on very few palms. However the present study involves exhaustive range of oil palm genotypes and populations for validation and confirmation of CAPS markers. Satish and Mohan Kumar [6] identified RAPD markers for identification of parental and hybrid genotypes. The identified CAPS marker is presently being utilized to group palms from a TXT cross into dura, pisifera and tenera groupings. We are also currently screening all the sprouts given to the farmers after screening for their fruit forms and we are also attempting to give tenera certification tag along with sprouts. Hence, the study will be useful in identifying the true female sterile pisifera palms, and also ensures supply of desired tenera sprouts to the oil palm growers instead of waiting for 4–5 years.

### Association mapping analysis

Association mapping analysis was conducted using the phenotypic data of eight oil yield related traits and the genotypic data of 50 microsatellite markers. Association of trait-marker data resulted in identification of seven significant QTLs by GLM approach, where as four significant QTLs were detected by MLM approach at a very significant threshold (P) level of 0.001 and 0.01 (Table 4). However, no QTLs identified for bunch weight and oil to wet mesocarp. This high cut-off level of significance was given to reduce the false QTLs which will give wrong interpretations. By GLM approach, two QTLs were identified for oil to bunch (%) trait linked by SPSC00033 and SPSC00067 SSR markers (Table 4). One QTL each linked to fruit to bunch (%), kernel to fruit (%), mesocarp to fruit (%), oil to dry mesocarp (%) and shell to fruit (%) traits. The oil to bunch trait was linked by two markers SPSC00033 and SPSC00067. Out of these two, SPSC00033 was significantly linked to the oil to bunch at P of 0.0002, explained R^2^ of 11.5%. Kernel to fruit and shell to fruit traits were found to be linked by SOTIG14040 and SMG00156 markers respectively. Interestingly, QTLs for mesocarp to fruit, oil to bunch, and shell to fruit traits were linked on chromosome 2, where the SHELL gene also located on the chromosome 2 [14]. Further fine mapping of the regions between shell gene and identified markers may be able to identify few SNPs related to shell thickness of oil palm.

**Table 4:** The details of the markers linked to eight oil yield related traits of oil palm.

Phenotypic trait	SSR Marker	Chromosome number	Probability of marker (*P*)	Phenotypic variance (R^2^) (%)	Sequence
**GLM approach**					
Fruit/Bunch (FB)	SPSC00178	14	0.0001	12.9	F5’TTTGGACTTGTCCATCCTCC3’ R5’TCTAGCTGCCAAAAGCTTGC3’
Kernel/fruit (KF)	SOTIG14040	12	0.009	10.3	F5’CCTCGAAGGTGAAGCAATAAAG3’ R5’ACTCATAGAGCTTTCCACGACC3’
Mesocarp to fruit (MF)	SPSC00067	2	0.002	7.5	F5’TACTTGATGCATAGGCTGCG3’ R5’AGGGTCATGAAATGTCGAACT3’
Oil/ bunch (OB)	SPSC00033	2	0.0002	11.5	F5’ATGGTCCCGTCCTAGGATTT3’R5’AACAGCTTGCCTCCTTGGTA3’
Oil/ bunch (OB)	SPSC00067	2	0.002	6.4	F5’TACTTGATGCATAGGCTGCG3’R5’AGGGTCATGAAATGTCGAACT3’
Oil/ dry mesocarp (ODM)	SPSC00185	14	0.006	6.8	F5’AAGGAGAACTACCACGCGAA3’R5’AATTATGTGCGGTTGTTGAGC3’
Shell/ fruit (SF)	SMG00156	2	0.002	5.1	F5’GGTGTCATAACTTCGTTGTTGCT3’ R5’ATGCTCAAAAGTGGGTTTCTCTC3’
**MLM approach**					
Oil/ bunch (OB)	SPSC00033	2	0.004	7.4	F5’ATGGTCCCGTCCTAGGATTT3’ R5’AACAGCTTGCCTCCTTGGTA3’
Fruit/Bunch (FB)	SPSC00178	14	0.001	9.6	F5’TTTGGACTTGTCCATCCTCC3’ R5’TCTAGCTGCCAAAAGCTTGC3’
Oil/ dry mesocarp (ODM)	SPSC00185	14	0.01	6.3	F5’AAGGAGAACTACCACGCGAA3’ R5’AATTATGTGCGGTTGTTGAGC3’
Shell/ fruit (SF)	SMG00156	2	0.01	5.1	F5’GGTGTCATAACTTCGTTGTTGCT3’ R5’ATGCTCAAAAGTGGGTTTCTCTC3’

By MLM approach, four significant QTLs were identified for oil to bunch, fruit to bunch, oil to dry mesocarp and shell to fruit traits. Significant associations were observed for oil to bunch (P- 0.004, R^2^–7.4%) and fruit to bunch (P- 0.001, R^2^ of 9.6%) by SPSC00033 and SPSC00178 markers respectively. The QTLs for oil to bunch (OB) and shell to fruit (SF) were linked by the markers SPSC00033 and SMG00156 respectively which were located on chromosome 2. Whereas by GLM approach, mesocarp to fruit also found to be located on the same chromosome along with oil to bunch and shell to fruit traits. Jeennor and Volkaert [24] identified QTLs for OB, and SF traits using SSRs by linkage mapping approach. In the present study QTLs for OB were identified on chromosome 2, whereas Jeennor and Volkaert [24] identified on chromosome 4 and 12. For SF trait, they found QTLs on chromosome 4, whereas present results identified on chromosome 2. The present results are more interesting, since the identified QTLs also located on chromosome 2 where the shell thickness gene also located. This paves the way for further fine mapping between shell gene and identified markers for identifying more SNPs or QTLs for shell thickness and oil yield traits. Since the whole genome sequence of oil palm is available, we searched for the homology of the markers on the reference genome. The marker (SOTIG14040) linked to kernel to fruit was showed homology to MADS box transcription factor 6 like iso-form X1. The CAPS marker used in the present study also obtained from the MADS box transcription factor 21 like sequence. The results of the study gives a scope for further study on the other transcription factors like the one identified in the study for their role in the shell thickness and oil to kernel which influences the oil yield. We are also further fine mapping the present results with more germplasm, SSR markers and SNPs by genotyping by sequencing approaches.

## Conclusion

The identified shell specific CAPS marker benefits the oil palm breeders and seed producers at several stages. The CAPS marker can use in certification of high yielding seed production by selective breeding of D X P crosses. It is useful where there is no stringent control on the pollination of oil palm plantations or natural pollinated plantations. It also useful to identify the true female sterile pisifera palms with out going for recurrent selection of T X T or T X P crosses. Supply of desired, high quality and yielding tenera sprouts to the oil palm growers was the prerequisite for any oil palm breeding programmes. In such instances, this single CAPS marker facilitates selection and distribution of desirable high yielding tenera sprouts to the farmers instead of waiting for 4–5 years. This saves a lot of land, time and money which will be a major breakthrough to the oil palm community. Four significant QTLs identified by GLM and MLM approaches can be used for further fine mapping and marker assisted selection for oil yield related traits in oil palm.
